# Comparative transcriptome analysis of the Asteraceae halophyte *Karelinia caspica* under salt stress

**DOI:** 10.1186/1756-0500-7-927

**Published:** 2014-12-17

**Authors:** Xia Zhang, Maoseng Liao, Dan Chang, Fuchun Zhang

**Affiliations:** Xinjiang Key Laboratory of Biological Resources and Genetic Engineering, College of Life Science and Technology, Xinjiang University, 14 Sheng li Road, Urumqi, 830046 China

## Abstract

**Background:**

Much attention has been given to the potential of halophytes as sources of tolerance traits for introduction into cereals. However, a great deal remains unknown about the diverse mechanisms employed by halophytes to cope with salinity. To characterize salt tolerance mechanisms underlying *Karelinia caspica*, an Asteraceae halophyte, we performed Large-scale transcriptomic analysis using a high-throughput Illumina sequencing platform. Comparative gene expression analysis was performed to correlate the effects of salt stress and ABA regulation at the molecular level.

**Results:**

Total sequence reads generated by pyrosequencing were assembled into 287,185 non-redundant transcripts with an average length of 652 bp. Using the BLAST function in the Swiss-Prot, NCBI nr, GO, KEGG, and KOG databases, a total of 216,416 coding sequences associated with known proteins were annotated. Among these, 35,533 unigenes were classified into 69 gene ontology categories, and 18,378 unigenes were classified into 202 known pathways. Based on the fold changes observed when comparing the salt stress and control samples, 60,127 unigenes were differentially expressed, with 38,122 and 22,005 up- and down-regulated, respectively. Several of the differentially expressed genes are known to be involved in the signaling pathway of the plant hormone ABA, including ABA metabolism, transport, and sensing as well as the ABA signaling cascade.

**Conclusions:**

Transcriptome profiling of *K. caspica* contribute to a comprehensive understanding of *K. caspica* at the molecular level. Moreover, the global survey of differentially expressed genes in this species under salt stress and analyses of the effects of salt stress and ABA regulation will contribute to the identification and characterization of genes and molecular mechanisms underlying salt stress responses in Asteraceae plants.

**Electronic supplementary material:**

The online version of this article (doi:10.1186/1756-0500-7-927) contains supplementary material, which is available to authorized users.

## Background

Salinity is a major environmental factor limiting plant growth and productivity. Thus, salt-tolerant halophytes serve as an excellent resource for the identification of desirable traits and the subsequent development of new crop systems [1]. Understanding the salt tolerance mechanisms in such plants represents an important step towards generating crop varieties capable of coping with environmental stresses. Towards this end, there is still much to learn about the diverse mechanisms employed by halophytes to cope with salinity stress.

*Karelinia caspica*, an herbaceous Asteraceae perennial that grows in saline deserts and swamps [2], has broad-spectrum resistance to pests and is also tolerant to salinity, drought, low temperatures, and high temperatures [3]. Due to its extreme desalination capacity, *K. caspica* is viewed as a good pioneer plant for the improvement of saline soil [4]. As a secretohalophyte, *K. caspica* actively absorbs and discharges salt through special glands and salt holes on the leaf surface [2, 3]. At the same time, salt stress promotes *K. caspica* succulence [2], which is typical of euhalophytes. Considering these mechanisms in aggregate, the salt tolerance of *K. caspica* as intermediate between that of euhalophytes and recretohalophytes is worth examining.

For most non-model organisms, the lack of whole-genome sequencing data continues to represent a major hurdle for research, as genome sequencing is still largely impractical for most eukaryotes and cannot be finished in a short timeframe. In contrast, high-throughput transcriptome analysis can be performed whether or not genomic sequences for the organism of interest are available [5] and thus represents a feasible approach for organisms that have not been sequenced. To date, transcriptome analysis has been employed in a wide range of eukaryotes including the following: yeast [6], mice [7], humans [8], Arabidopsis [9], *Caenorhabditis elegans* [10], rice [11], and *Vitis vinifera* [12].

In addition to regulating diverse aspects of plant growth and development, abscisic acid (ABA) is also required for plant resistance to drought and salt stress [13–15]. Numerous studies have aimed to elaborate the cellular and molecular responses of plants to ABA, including those related to ABA sensing, signaling, metabolism, and transport.

To globally survey the salinity-induced ABA responses in *K. caspica*, RNA-Seq technology was used. The transcription profiles of control and salt-stressed plants were compared, and dynamic changes in the transcriptome were analyzed. The goal of the present work was to elucidate physiological processes, including those involved in the ABA regulatory network, that are induced by salt stress in the halophyte *K. caspica* at the transcriptomic level.

## Methods

### Plant materials

The *K. caspica* seeds used in this study were obtained from saline land (Wujiaqu, Xinjiang, China). Specific permission was not required for this collection activity at these locations, which are considered wastelands, and the field studies did not involve endangered or protected species. Eight-week-old seedlings germinated in perlite:vermiculite substrate (3:1) supplemented with 300 mM NaCl at 25°C and grown under a 16-hour photoperiod were used for the expression analysis. The treated and non-treated groups were sampled at 3, 6, 12, 24, 36, and 48 hours. After washing with deionized distilled water, the material from the different time points was combined to generate the final samples used for cDNA library preparation.

### Preparation of cDNA Libraries for RNA-Seq

For each treatment (i.e., the control and salt stress treatments), approximately 100 g fresh material was used for RNA preparation. Total RNA was extracted using the RNAqueous Kit [16] then subsequently treated with RNase-free DNase I (QIAGEN #79254) to remove residual genomic DNA. For each treatment, mRNAs were treated with Truseq RNA sample preparation kit (Illumina-15026495, USA), firstly purified from the 20 mg total RNA using oligo (dT) magnetic beads and fragmented using fragmentation buffer. Cleaved short RNA fragments were used for first-strand cDNA synthesis using reverse transcriptase and hexamer primer, followed by second strand cDNA synthesis using DNA polymerase I and RNase H. Following a quality check using an Agilent 2100 Bioanalyzer, the cDNA libraries were used for sequence analysis using the Illumina HiSeq TM 2000 system.

### Transcriptome analysis

Raw sequencing data were deposited in the DDBJ/EMBL/GenBank database (accession number GANI00000000). After the adapter sequences were trimmed from the raw reads, empty reads and reads containing unknown nucleotides (Ns) > 5 were removed. The remaining clean reads were *de novo* assembled into unigenes using Trinity software (trinityrnaseq_r2012-06-08 edition). The paired-end method was then used to acquire a single set of non-redundant unigenes. Glimmer 3.02 and EMBOSS_6.3 software were used to analyze the coding sequences (CDS) of the unigenes. All non-redundant unigenes were used to perform BLAST searches and to obtain annotation information in the following databases: NCBI nr, Swiss-Prot, Kyoto Encyclopedia of Genes and Genomes (KEGG), and Cluster of Orthologous Groups (KOG). WEGO [17] was used to classify GO function, which was analysis using the Blast2go program [18].

### Identification of differentially expressed genes

RPKM (reads per kilobase per million reads) values were used to evaluate expression and to quantify transcript levels [19], and differentially expressed unigenes [20] were identified by fold change values and fisher test. In the present study, the fold.change (AB) value ≥ 1 were choosen was used to as the threshold to determine significant differences in gene expression. For the pathway enrichment analysis, all differentially expressed unigenes were mapped to terms in the KEGG database. A search was then performed for significantly enriched KEGG terms compared to the whole transcriptome background.

### Quantitative RT-PCR (qRT-PCR) analysis

For qRT-PCR, total RNA was isolated from 200 mg frozen materials as described above. Three biological replicates were performed for both the control and salt stress-treated seedlings. For each qRT-PCR reaction, 100 ng of RNA that had been treated to remove genomic DNA was used as template for cDNA synthesis. Additional file 1: Table S1 lists the sequences of the primer pairs used for qRT-PCR. The relative transcript levels of selected genes were quantified using Platinum® SYBR® Green qPCR SuperMix-UDG (Invitrogen, CA, USA). The reactions were performed using the 7500 Real-Time PCR system and software (Applied Biosystems, CA, USA). *KcACTIN*, which was constant under the tested conditions, was included for normalization in each qRT-PCR run. Average expression ratios (PQ/C) were calculated using the ΔΔCT method, and log2 fold-change values were used.

## Results and discussion

### Raw reads processing and assembly

To obtain transcript information for *K. caspica*, two cDNA libraries were prepared for the control and salt stress-treated plants then sequenced using the Illumina sequencing platform. The raw reads were transformed by base calling from the image data output from sequencing. After trimming the adapter sequences and removing sequences with unknown or low quality bases, approximately 50.35 and 51.58 million clean reads were obtained for the control and salt stress samples, respectively. Trinity software (trinityrnaseq_r2012-06-08) and the paired-end method were used for *de novo* assembly of the clean reads into contigs. A total of 287,185 transcripts with different lengths were generated from both of the treatments (Additional file 2: Table S2), which provided abundant information for subsequent analysis of salt stress-associated genes in *K. caspica*.

### Functional annotation and GO assignments of the assembled transcripts

**Figure 1:**
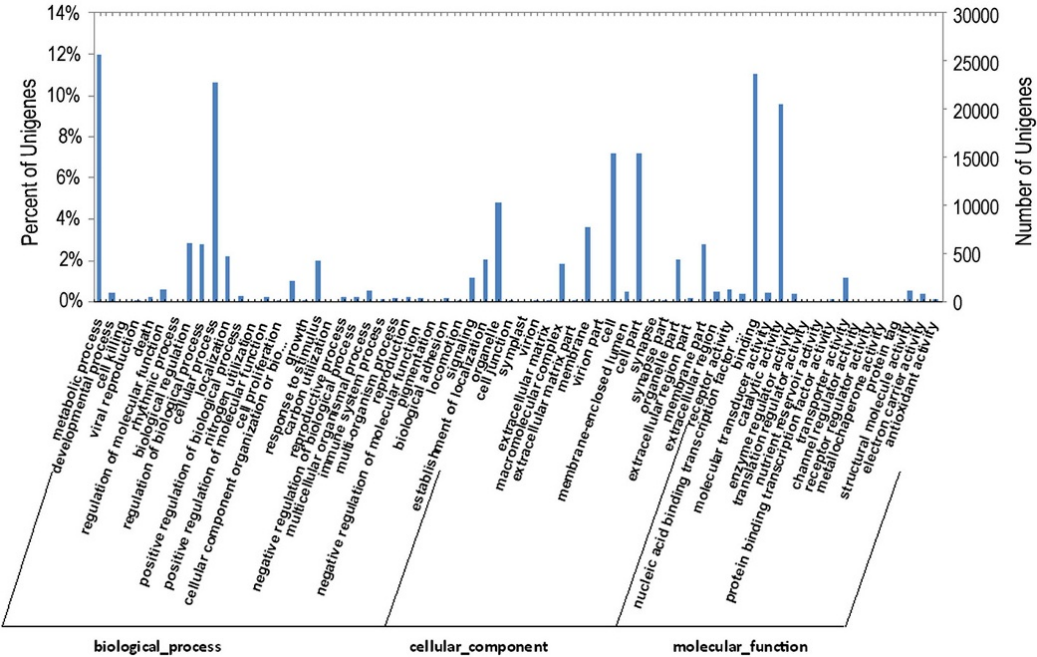
**Diagram of the KOG (clusters of orthologous groups) classification.** A total of 18,378 sequences were classified under 25 KOG categories. All of the unigenes were grouped into three major functional categories: biological process, cellular component, and molecular function. The right y-axis indicates the number of unigenes in a given category. The left y-axis indicates the percentage of a given category within the main category.

**Figure 2:**
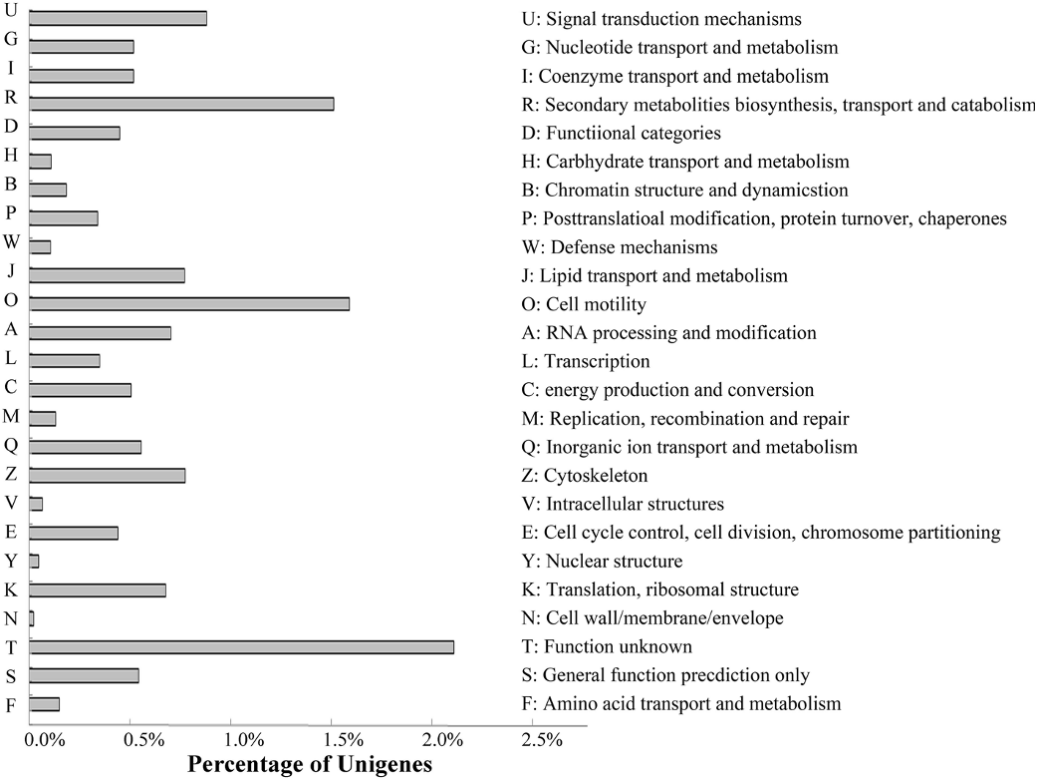
**GO annotation of non-redundant unigenes.** Good hits were aligned to the GO database, and 35,533 transcripts were assigned to at least one GO term.

The unigene sets obtained from the *K. caspica* transcriptome data were annotated based on protein sequence homology. First, we used glimmer3.02 and EMBOSS_6.3.1 software to transform all transcripts with sequences longer than 100 bp into reliable coding sequences (CDS). The 320,260 CDS produced were used for further annotation with the NCBI nr, Swiss-Prot, TrEMBL, CDD, pfam, and KOG databases. From this analysis, 216,415 unigenes were identified that exhibited high sequence similarity with known gene sequences (Additional file 3: Table S3 and Additional file 4: Table S4). Subsequently, a total of 18,378 sequences were assigned to 25 KOG (clusters of orthologous groups for eukaryotic complete genomes) terms. Among the terms identified, the three highest represented categories were as follows: signal transduction mechanisms (4,561); posttranslational modification, protein turnover, chaperones (3,441); and general function prediction (3,271) (Figure 1). All of the unigenes were queried in the GO database (Gene Ontology, an international standardized gene functional classification) to classify their predicted functions. From this analysis, 35,533 unigenes were grouped into three functional categories, "biological process", "cellular component", and "molecular function", with the subsets of sequences further divided into 33, 18, and 17 subcategories in these three groups, respectively. The largest subcategory in the "biological process" group was "metabolic process", which included 12.1% of the unigenes in the subcategory. In the "cellular component" and "molecular function" categories, "cell" and "binding activity" were the most abundant GO terms, representing 7.5% and 11.3% of each subcategory, respectively. In addition, there were high percentages of unigenes in the "cell part", "catalytic activity", and "cellular process" categories and only a few unigenes in the "receptor regulator activity", "locomotion", "cell junction", and "symplast" categories (Figure 2).

### Changes in gene expression under salt stress

To investigate the molecular response to salt stress exposure, unigene transcript levels in the control and salt stress treatments were calculated as RPKM (reads per kilobase per million reads), which eliminates the influence of gene length and sequencing discrepancy in calculating gene transcript levels and permits a direct comparison between treatments [19]. Based on the RPKM values observed, 60,127 differentially expressed unigenes were detected (Additional file 5: Table S5), with 38,123 and 22,005 up- and down-regulated genes, respectively. Salinity imposes a water deficit and ion stress, which have wide-ranging effects on the activity of plant cells, including inhibition of essential enzymes, cell membrane destabilization, a decrease in nutrient supply, and overproduction of reactive oxygen species (ROS) [15, 21]. The extensive variation observed in the transcriptome (67.3%) indicates complex transcriptional changes in *K. caspica* and comprehensive salt-stress influence on the cellular activity of *K. caspica*.

### Functional annotation of differentially expressed unigenes under salt stress

**Figure 3:**
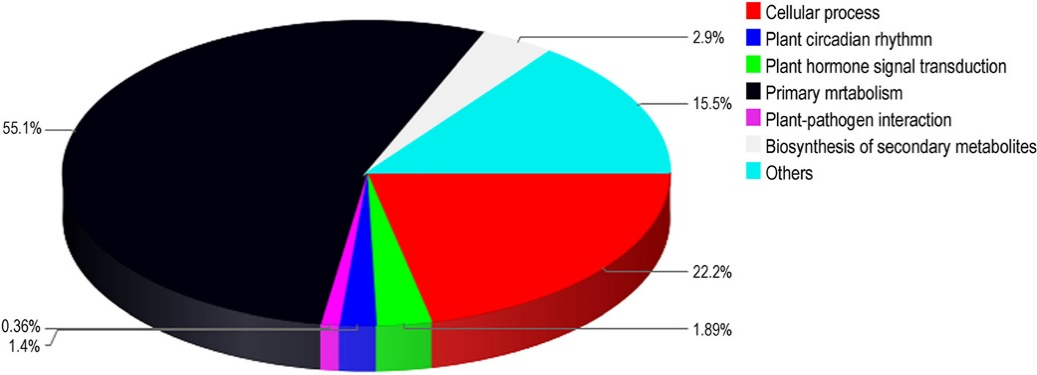
**KEGG functional analysis of the differentially expressed unigenes.**

To identify unigenes involved in metabolic or signal transduction pathways that were significantly enriched under salt stress, all of the differentially expressed sequences were queried in the KEGG database and compared to whole transcriptome data. Among the 60,127 differentially expressed transcripts (DETs), 13,848 genes were well annotated, while the remaining 46,239 genes had low sequence similarity to known sequences in the current database and therefore represent potentially novel salt-stress responsive genes. The potentially large number of unknown regulated genes suggested that factors involved in salt stress responses in Asteraceae may be distantly related to those identified in other genera. Pathway enrichment analysis revealed that many genes for which annotation data were available were directly or indirectly involved in the salt stress response, namely, primary metabolism, cellular processes, plant hormone signal transduction, plant-pathogen interaction, biosynthesis of secondary metabolism, and plant circadian rhythm (Figure 3 and Additional file 6: Table S6). These findings underscore the large scale re-coordination that occurs during short-term acclimation to salt stress exposure. Among the 4,023 DETs with pathway annotation, 172 DETs were found to be involved in the plant hormone signal transduction after salinity exposure, and 68 DETs were associated with MAPK signaling pathway (Table 1). Since very little information about the signaling cascades and the pathway of salinity sensing in Asteraceae is available, the presently identified sequences provide important clues for screening these putative salt stress responsive genes and their associated genes.

**Table 1:** **Significantly enriched pathways of differentially expressed unigenes**

Pathway category	Unigenes No	%	Qvalue
Plant hormone signal transduction	172	4.28	1.59E-14
MAPK signaling pathway	68	1.69	1.45E-19
Calcium signaling pathway	32	0.80	1.29E-14
Nitrogen metabolism	29	0.72	2.61E-14
Circadian rhythm	20	0.50	2.61E-14
Steroid hormone biosynthesis	11	0.27	8.92E-12

Unigenes No. and % indicate the number and the percentage of unigenes in each pathway from 4023 differentially expressed unigenes mapped to KEGG respectively.

### Functional annotation of differentially expressed unigenes involved in ABA-signaling under salt stress

ABA plays a key role in a wide range of developmental processes and adaptive stress responses to environmental stimuli in plants [22]. Many studies have focused on the cellular and molecular responses in plants to ABA, including ABA metabolism, transport, sensing, and signaling. We therefore focused on these physiological processes and highlighted potentially informative findings determined from the analysis.

### ABA metabolism

ABA is synthesized from carotenoids. 9-cis-epoxycarotenoid dioxygenase (NCED) breaks down the 11, 12 double bond of 9-cis violaxanthin, which results in the formation of C_15_ xanthoxin within plastids. The subsequent steps of xanthoxin conversion to abscisic aldehyde are catalyzed by abscisic-aldehyde oxidase (AAO3) and xanthoxin dehydrogenase (ABA2) in the cytosol [23]. In the salt-stressed *K. caspica* transcriptome, NCED (comp39466), AAO3 (comp43593), and ABA2 (comp714908) were all identified as up-regulated DETs. Consistent with this finding, carotenoid cleavage dioxygenase (CCD, comp40565), which yields another carotenoid-derived phytohormone from carotenoid and therefore competes with NCED for the same substrate, was down-regulated, further implicating enhanced ABA content in the responses of *K. caspica* to salt stress.

### ABA transport

Although ABA is predominantly biosynthesized and metabolized in vascular tissues, it acts in the stomatal responses of distant guard cells [24–26]. In this way, ABA intercellular regulation and transport are critical for plant responsiveness to osmotic stress. It was recently reported that two G-type ATP-binding cassette (ABC) transporter genes, AtABCG25 and AtABCG40, encode proteins responsible for ABA transport and response in Arabidopsis [27]. The ABC transporter is conserved in many model species from *E. coli* to humans and was reported to transport various metabolites and signaling molecules through the action of phytohormones in an ATP-dependent manner [28, 29]. Several different types of ABC transporters (Table 2) were identified as up-regulated DEGs (differentially expressed genes) in *K. caspica* through BLAST homology searches, but only one unigene (comp306780) belonged to the G-type ABC subfamily. Nevertheless, this finding does not preclude the possibility that other non-G-type ABC transporters or non-ABC transporters identified in *K. caspica* contribute to cell-to-cell ABA vesicular transport. In fact, two nitrate transporter 1/peptide transporters (NRT1/PTR) family members involved in the transport of nitrogen (N) compounds were recently characterized as novel ABA transporters in Arabidopsis despite being wholly distinct from ABC transporter family members [23].

**Table 2:** **Putative ATP-binding cassette (ABC) transporter genes identified as up-regulated DEGs in** ***K. caspica***

Unigene ID	Length (bp)	Subfamily	Best hit	E-value	Blast annotation/Organism
comp465528	297	A	Q99758	1E-63	ATP-binding cassette sub-family A member 3 [*Homo sapiens*]
comp16398	388	B	Q8LPT1	3E-54	ABC transporter B family member 6 [*Arabidopsis thaliana*]
comp17267	328	B	Q9ZR72	1E-59	ABC transporter B family member 1 [*Arabidopsis thaliana*]
comp450357	451	B	Q704E8	2E-61	ATP-binding cassette sub-family B member 7 [*Rattus norvegicus*]
comp454701	282	B	Q8LPK2	7E-15	ABC transporter B family member 2 [*Arabidopsis thaliana*]
comp577194	451	B	Q9NRK6	3E-17	ATP-binding cassette sub-family B member 10 [*Homo sapiens*]
comp20933	300	C	Q9LZJ5	9E-47	ABC transporter C family member 14 [*Arabidopsis thaliana*]
comp267971	705	D	P48410	9E-125	ATP-binding cassette sub-family D member 2-like [*Acyrthosiphon pisum*]
comp324163	542	D	P48410	4E-40	ATP-binding cassette sub-family D member 1 [*Mus musculus*]
comp352820	476	D	Q9QY44	9E-45	ATP-binding cassette sub-family D member 2 [*Rattus norvegicus*]
comp5337	228	D	Q9UBJ2	3E-20	ATP-binding cassette sub-family D member 2 [*Homo sapiens*]
comp481971	244	E	P61222	5E-30	ATP-binding cassette sub-family E member 1 [*Mus musculus*]
comp29933	2612	F	Q9UG63	0.0	ATP-binding cassette sub-family F ember 2 [*Tribolium castaneum*]
comp516400	351	F	Q8K268	2E-33	ATP-binding cassette sub-family F member 2 [*Homo sapiens*]
comp306780	297	G	Q9H172	1E-46	ATP-binding cassette sub-family G member 4 [*Homo sapiens*]

### ABA sensing and signaling

**Figure 4:**
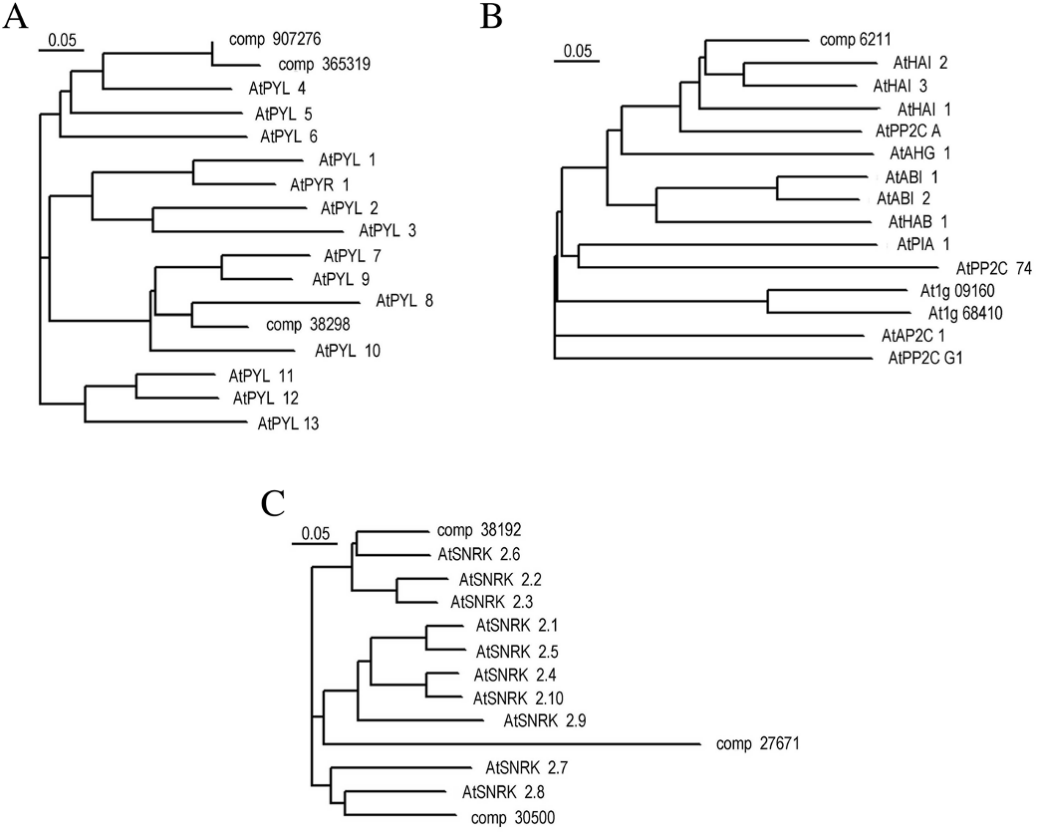
**Sequence alignment and phylogenetic trees of (A) PYL, (B) PP2C, and (C) SnRK2 gene family members in** ***Arabidopsis thaliana*** **along with DEGs identified in** ***K. caspica*** **.** Sequence data referenced here can be found in the GenBank data libraries under the following accession numbers: PYL1 (AT5G46790), PYL2 (AT2G26040), PYL3 (AT1G73000), PYL4 (AT2G38310), PYL5 (AT5G05440), PYL6 (AT2G40330), PYL7 (AT4G01026), PYL8 (AT5G53160), PYL9 (AT1G01360), PYL10 (AT4G27920), PYL11 (AT5G45860), PYL12 (AT5G45870), PYL13 (AT4G18620), PYR1 (AT4G17870), SNRK2.1 (AT5G08590), SNRK2.2 (AT3G50500), SNRK2.3 (AT5G66880), SNRK2.4 (AT1G10940), SNRK2.5 (AT5G63650), SNRK2.6 (AT4G33950), SNRK2.7 (AT4G40010), SNRK2.8 (AT1G78290), SNRK2.9 (AT2G23030), SNRK2.10 (AT1G60940), PP2CA (AT3G11410), HAI1(AT5G59220), AHG1(AT5G51760), PP2CG1(AT2G33700), AP2C1(AT2G30020), HAI2(AT1G07430), PIA1(AT2G20630), HAI3(AT2G29380), PP2C74(AT5G36250), AtHAB1(AT1G72770), AtABI2(AT5G57050), and AtABI1(AT4G26080).

In one proposed model of ABA signaling in Arabidopsis, PYR/RCAR/PYL (Pyrabactin Resistance/Regulatory Component of ABA Receptor/Pyrabactin Resistance 1-Like) family proteins, which act as ABA receptors, recognize and bind to group A PP2C (type 2C protein phosphatase) molecules in the presence of ABA. Subclass III SnRK2s (SNF1-related protein kinase 2) are then released from PP2C-dependent negative regulation, allowing the activated SnRK2s to phosphorylate downstream proteins such as ABA-responsive element (ABRE)-binding transcription factors (ABFs) [30–32]. Similar regulation of ABA signaling has been detected in other species such as wheat [30, 33, 34]. In *K. caspica*, orthologs of PYL (comp365319, comp38298), PP2C (comp6211), and SnRK2 (comp 30500, comp27671, comp38192) were up-regulated in the salt stress sample (Figure 4), indicating that the regulation of ABA signaling is indeed conserved among plant species.

Based on phylogenetic analysis, the DEGs comp365319, comp38298, and comp907276 in *K. caspica* grouped together with AtPYL4-9 (Figure 4). Since AtPYL4, AtPYL5, AtPYL6, AtPYL8, and AtPYL9 have been found to inhibit PP2Cs even in the absence of ABA [35] and considering that the ectopic expression of PYL5 and PYL8 in Arabidopsis results in enhanced drought resistance [36, 37], comp365319, comp38298, and comp907276 may function independently of ABA as positive regulators in the salinity response of *K. caspica*. Moreover, AtHAI1, AtHAI2, and AtHAI3 (Highly ABA-Induced PP2Cs) and the DEG comp6211 in *K. caspica* made up the clade A PP2Cs (Figure 4), which had the greatest effect on ABA-independent low water potential phenotypes and less of an effect on classical ABA sensitivity phenotypes [38]. Thus, comp6211 was associated with known clade A PP2Cs in ABA-independent salinity-associated signaling. Furthermore, physiological analyses illustrated that the DEGs comp27671, comp30500, and comp38192 were classified into groups I, II, and III, respectively (Figure 4). Of these, only group III SnRKs are considered ABA-dependent kinases [39, 40]. It nevertheless remains possible that many of the *K. caspica* DEGs may be involved in ABA-independent salinity-signaling cascades.

### Real-time PCR validation of differentially expressed unigenes

**Figure 5:**
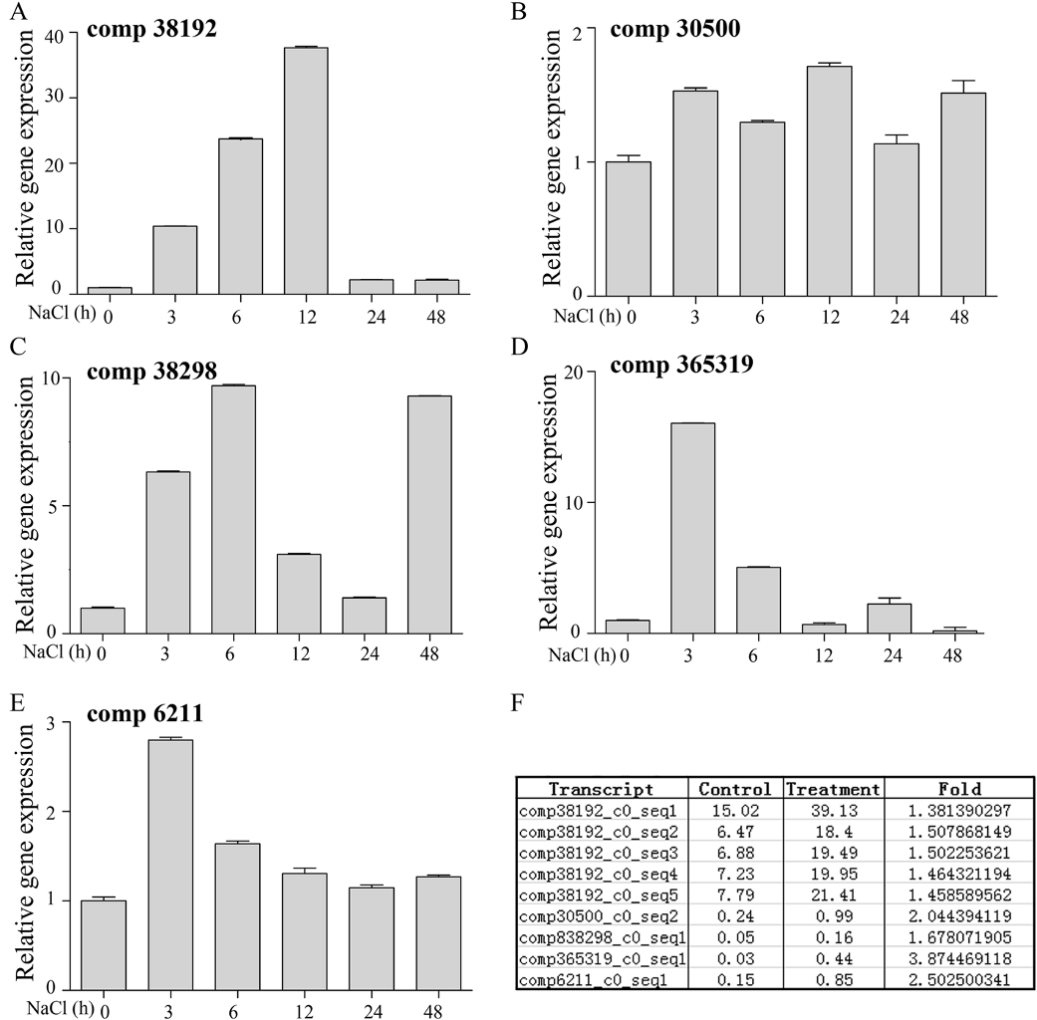
**Real-time qRT-PCR analysis of selected genes from the RNA-seq dataset.** Total RNA was extracted from *K. caspica* exposed to salt stress, and real-time PCR was used to validate the changes in gene expression of putative SnRK2 (comp38192, **A**; comp 30500, **B**), PYL (comp38298, **C**; comp365319, **D**), and PP2C (comp6211, **E**). *KcACTIN* was used as the internal control. Error bars indicate standard deviations from three independent biological samples. And the fold change estimated by transcriptomic ananlysis were also illustrated in **F**.

To validate the transcriptome data of K. caspica under salt stress, five DEGs that were found to be up-regulated in K. caspica exposed to salt stress, namely, orthologs of SnRK2 (comp38192, comp 30500), PYL (comp38298, comp365319), and PP2C (comp6211), were selected for real-time PCR analysis using two-month-old K. caspica seedlings treated with 300 mM NaCl. As shown in Figure 5, all five genes exhibited enhanced expression at certain points during the treatment period, confirming the comparative transcriptome data for salt-stressed K. caspica. Comp6211 and comp30500, which are predicted to be ABA-independent signaling components, also changed over the course of the treatment period, implicating their functions in K. caspica responses to salinity. As ABA receptors, PYL (comp38298, C; comp365319, D)responded earlier in the presence of salinity stress, recognized and bind to group A PP2C (comp6211, E) molecules. Subclass III SnRK2s (comp38192, A; comp 30500, B), which responded to salinity stress at 12 h, a little later than PYL (comp38298, C; comp365319, D) and PP2C (comp6211, E), are then activated, allowing the SnRK2s to phosphorylate downstream proteins. And the expression of the five genes was comparable with the fold change estimated by transcriptomic data illustating that the changes presented in real-time PCR are biologically significant. Taken together, these results validated the involvement of ABA in K. caspica subjected to salt stress. Actually, ABA is universal as stress-invlolved hormone in plant kimdom. Besides, the core components in ABA signaling have been obtained in rice, *Selaginella moellendorffi*, *Physcomitrella patens*, *Ostreococcus tauri* [41]. Although the PYR/PYL/RCAR–PP2C–SnRK2 pathway model has been established [41], it is not clear whether this model can explain all ABA responses in plant kindom. It is necessary to determine whether these redundant variants are dependent on or independent of the core ABA pathway among different plants.

### Other DGEs involved in other hormone signaling

Plants adapt to adverse environments by integrating growth and development to environmentally activate cues. Besides ABA, several DGE involved in other hormone signaling indicated multiple hormone crosstalks in *K.caspica* responses to salt stress. For example, GA integrates generic responses into abiotic stress tolerance via the DELLA proteins [42] (comp30323, comp47577) to regulate plant development. Auxin modulate plant development,especially root system architecture, to defense for salt stress by SAUR family protein (comp 31574, comp181105, etc.) and auxin responsive GH3 gene family (comp324952, comp9514, etc.) [43, 44]; Brassinosteroid (BR) and ethylene, which respectively mediated by BR-signaling kinase (comp768925), ethyle-reponsive transcription factor (ERF1, comp20087; ERF2, comp382497), and EIN3-binding F-box protein (EBF1-2, comp768925), are also involved in strateges plants take to cope with unfavorable conditions [45, 46]. Therefore, the response to salt stress in *K.caspica* is a comprehensive regulatory network in term of the plant hormones signaling cascade, and need further elucidated.

## Conclusion

This study profiled the transcriptome of *K. caspica* under salt stress using Illumina RNA-seq technology to identify responsive genes and specific pathways involved in the salinity response of *K. caspica*. The transcriptome profile data provide a foundation for further investigation of the molecular basis underlying salt stress tolerance in this species. Several key genes involved in ABA metabolism, transport, sensing, and signaling functions were found to be induced by salt stress treatment. Thus, the transcriptome profiling approach and subsequent gene expression analysis in *K. caspica* provide important clues for the identification of functional genes and the contribution of the ABA signaling pathway to the salt tolerance of Asteraceae plants.

## Electronic supplementary material

Additional file 1: Table S1: Primer pairs used for real-time. (DOCX 16 KB)

Additional file 2: Table S2: Overview of de novo assembly statistics for K. caspica transcriptome sequencing. (DOC 28 KB)

Additional file 3: Table S3: Detailed information for 216,415 unigenes annotated in the K. caspica transcriptome. (ZIP 16 MB)

Additional file 4: Table S4: Overview of unigene annotation statistics collected from the NCBI nr, Swiss-Prot, TrEMBL, CDD, pfam, and KOG databases. (DOC 30 KB)

Additional file 5: Table S5: Detailed information for 60,127 differentially expressed unigenes between the control and salt stress samples. (XLSX 11 MB)

Additional file 6: Table S6: KEGG functional analysis of the differentially expressed unigenes. (XLSX 22 KB)
