# Supplementary material for: Comparative transcriptome analysis of the Asteraceae halophyte Karelinia caspica under salt stress
Source: BMC Res Notes. 2014 Dec 17;7:927. doi: 10.1186/1756-0500-7-927 (PMC4320537; doi:10.1186/1756-0500-7-927)
Supplement: Supplementary file 1 — Additional file 1: Table S1: Primer pairs used for real-time. (DOCX 16 KB) [file 13104_2013_3468_MOESM1_ESM.docx]

**Table S1**  Primer pairs used for realtime-PCR

| Gene | Primer |
| --- | --- |
| comp6211 | 5’- GCTTGTTGGTTCCAGGTTTTTA -3’ |
|  | 5’- AAAGATTACACATCTTGGTGAAGG -3’ |
| comp38298 | 5’- GTGTTTGACGACAGCGGAAG -3’ |
|  | 5’- GGTACACACGCTATTTTCATCAC -3’ |
| comp365319 | 5’- TCCACAATCAGTTTACCTCCCT -3’ |
|  | 5’- GATCACCAGGACCTCTCTTATTG -3’ |
| comp38192 | 5’- ATCGTAGCGGTCACTGTCGT -3’ |
|  | 5’- TCCTATGCGGTAGCCGAGTC -3’ |
| comp30500 | 5’- GGCTACCCCAAAATTACCAGA -3’ |
|  | 5’- CCACAAAACACTTCATCATCTACC -3’ |
| *KcActin* | 5’- AGGTCACGACCAGCAAGATCA -3’ |
|  | 5’- TGCTGGATTCTGGAGATGGTG -3’ |
