# Supplementary material for: Comparative transcriptome analysis of the Asteraceae halophyte Karelinia caspica under salt stress
Source: BMC Res Notes. 2014 Dec 17;7:927. doi: 10.1186/1756-0500-7-927 (PMC4320537; doi:10.1186/1756-0500-7-927)
Supplement: Supplementary file 2 — Additional file 2: Table S2: Overview of de novo assembly statistics for K. caspica transcriptome sequencing. (DOC 28 KB) [file 13104_2013_3468_MOESM2_ESM.doc]

Table S2. Overview of *de novo* assembly statistics on K.caspica transcriptome sequencing.

|  | All (>=100bp) | >=500 bp | >=1000 bp | N50 | N90 | Total Length | Max Length | Min Length | Average Length |
| --- | --- | --- | --- | --- | --- | --- | --- | --- | --- |
| Transcript | 287185 | 92108 | 58279 | 1599 | 220 | 187439691 | 18619 | 101 | 652.68 |
