# Supplementary material for: Comparative transcriptome analysis of the Asteraceae halophyte Karelinia caspica under salt stress
Source: BMC Res Notes. 2014 Dec 17;7:927. doi: 10.1186/1756-0500-7-927 (PMC4320537; doi:10.1186/1756-0500-7-927)
Supplement: Supplementary file 4 — Additional file 4: Table S4: Overview of unigene annotation statistics collected from the NCBI nr, Swiss-Prot, TrEMBL, CDD, pfam, and KOG databases. (DOC 30 KB) [file 13104_2013_3468_MOESM4_ESM.doc]

Table S4. Overview of unigene annotation statistics with with the nr, SWISS-PROT, TrEMBL, Cdd, pfam, and KOG databases

| Unigene_num | NR | SWISS-PROT | TREMBL | CDD | PFAM | KOG |
| --- | --- | --- | --- | --- | --- | --- |
| 216415 | 44145 | 27945 | 44791 | 24000 | 39079 | 18378 |
|  | 20.40% | 12.91% | 20.70% | 11.09% | 18.06% | 8.49% |
